# Controllable Preparation and Strengthening Strategies towards High-Strength Carbon Nanotube Fibers

**DOI:** 10.3390/nano12193478

**Published:** 2022-10-05

**Authors:** Yukang Zhu, Hongjie Yue, Muhammad Junaid Aslam, Yunxiang Bai, Zhenxing Zhu, Fei Wei

**Affiliations:** 1 Beijing Key Laboratory of Green Chemical Reaction Engineering and Technology, Department of Chemical Engineering, Tsinghua University, Beijing 100084, China; 2 CAS Key Laboratory of Nanosystem and Hierarchical Fabrication, CAS Center for Excellence in Nanoscience, National Center for Nanoscience and Technology, Beijing 100190, China

**Keywords:** carbon nanotubes, carbon nanotube fibers, tensile strength, defect control, controlled preparation

## Abstract

Carbon nanotubes (CNTs) with superior mechanical properties are expected to play a role in the next generation of critical engineering mechanical materials. Crucial advances have been made in CNTs, as it has been reported that the tensile strength of defect-free CNTs and carbon nanotube bundles can approach the theoretical limit. However, the tensile strength of macro carbon nanotube fibers (CNTFs) is far lower than the theoretical level. Although some reviews have summarized the development of such fiber materials, few of them have focused on the controllable preparation and performance optimization of high-strength CNTFs at different scales. Therefore, in this review, we will analyze the characteristics and latest challenges of multiscale CNTFs in preparation and strength optimization. First, the structure and preparation of CNTs are introduced. Then, the preparation methods and tensile strength characteristics of CNTFs at different scales are discussed. Based on the analysis of tensile fracture, we summarize some typical strategies for optimizing tensile performance around defect and tube–tube interaction control. Finally, we introduce some emerging applications for CNTFs in mechanics. This review aims to provide insights and prospects for the controllable preparation of CNTFs with ultra-high tensile strength for emerging cutting-edge applications.

## 1. Introduction

Materials are the basis of the evolution of human civilization. The pursuit of the ultimate properties of materials, such as super strength and super toughness, has strongly promoted the development of human culture. In 1895, Konstantin Tsiokovsky, a Soviet scientist, put forward building a “sky castle” at the top of a giant tower, which later evolved into the concept of “space elevator”. By connecting the earth and the space station with a cable, people can achieve space sightseeing and transport items to the space station [1]. However, the biggest challenge of this concept is finding light and strong cable that can overcome its gravity. A variety of nanostructures can be composed of single carbon elements, such as fullerenes (0D), carbon nanotubes (1D), and graphene (2D). Carbon nanotubes (CNTs) are cylinders rolled from single or multi-layer graphene sheets. Single-walled carbon nanotubes (SWCNTs) are cylinders rolled from a single-layer graphene sheet, while double-walled carbon nanotubes (DWCNTs) and multi-walled carbon nanotubes (MWCNTs) are composed of two and multiple layers of rolled graphene sheets, respectively. As one of the strongest chemical bonds in nature [2,3], the in-plane σ covalent bond of graphene formed by sp^2^ hybridization endows CNTs with extremely high axial Young’s modulus (~1.1 TPa) and tensile strength (~120 GPa). Theoretical calculations have shown that CNTs are the most probable material to help mechanical materials achieve a breakthrough and even realize the “space elevator” dream [1]. However, CNTs with extremely excellent mechanical properties are nanoscale solids, and practical applications require macro-scale materials. It is the prerequisite for CNTs to play a significant role in practical applications that they can maintain excellent mechanical properties after being assembled from a single nanoscale unit to a macroscopic aggregate.

**Figure 1:**
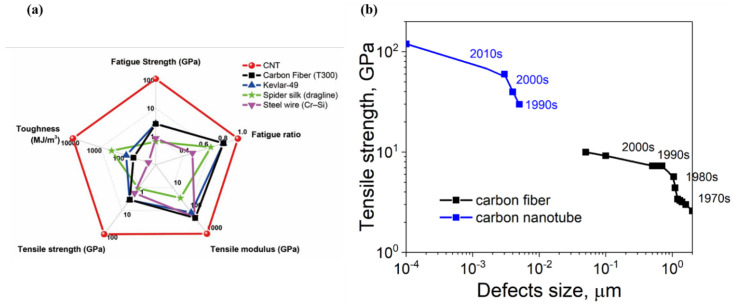
(**a**) Comparison of mechanical performance between CNTs and some high−performance materials. Reproduced with permission from [15]. Copyright 2020, American Association for the Advancement of Science. (**b**) Evolution of the tensile strength of CNTs and CFs at different defect sizes. The data are collected in [4,7,10,15].

In recent years, rapid progress has been made in the preparation and mechanical properties optimization of carbon nanotube fibers (CNTFs). Different spinning methods of CNTFs have been put forward and improved upon, and CNTFs with a tensile strength comparable to carbon fibers (CFs) have been prepared [4,5,6,7]. However, their mechanical properties are still far lower than single CNTs [7], which also shows an unsatisfactory phenomenon of property transfer across scales. Theoretical calculations and experimental results show that the tensile strength of CNTs with a nanoscale diameter can exceed 100 GPa [8,9]. CNT bundles with a diameter of 10–100 nm can have a tensile strength of up to 80 GPa [10]. CNTFs with a diameter of more than 1 μm, as a representative of macro assemblies of CNTs, have a maximum tensile strength of only 9.6 GPa [4], which is far lower than the intrinsic mechanical strength of CNTs. The reasons for such cross-scale tensile strength transfer are mainly due to defect accumulation and the lack of ideal tube–tube interactions during CNT assembly. Defects can have a fatal effect on the strength of CNTs [11,12]. With the increase in fiber size, defects also accumulate across scales. As shown in Figure 1b, the improvement of strength for CFs and CNTFs is closely related to the reduction in defect size [13,14]. For CFs, the tensile strength was increased from about 1 GPa to 10 GPa when the defect size was reduced from the micron to the nano scale. For CNTs, due to the fewer defects in structure compared with CFs, less attention was paid to their precise structural control, especially defects, resulting in their tensile strength having long been at a lower level. Until the 2000s, a series of achievements were made in the prepration of defect-free CNTs, which have shown extraodinary tensile strength performance both at the single-tube and bundle levels [9,15]. Therefore, the preparation of ideal solids such as defect-free or defectless CNTs is the basis for preparing CNTFs with high tensile strength. At the same time, many studies have shown that the mechanical properties of CNTFs can be significantly influenced by the tube–tube interactions involving orientation, length, and density. It is of great significance to regulate the tube–tube interactions and precisely control the atomic defects for the improvement of CNTs’ mechanical tensile strength [4,16,17,18].

In the past few decades, CNTFs have attracted extensive attention from academia and industry, and there are many reviews on the development and application of CNTFs [19,20,21,22,23]. However, few reviews have focused on the controllable preparation and strength optimization of CNTFs at different scales, which will be specifically highlighted in this review. First, the intrinsic mechanism of the excellent mechanical properties of CNTs and the preparation of CNTs are introduced. Then, the development and characteristics of techniques for fabricating CNTFs at different scales are discussed. Furthermore, we introduce the tensile strength of CNTFs from the nanoscale to the microscale, showing the recent development in the transfer of tensile properties across scales. Then, we analyze strategies for the strength optimization of CNTFs at different scales, particularly the aspects of defect and tube–tube interaction control. Finally, we provide an outlook for the practical applications of CNTFs with high mechanical strength. As a result, we aim to provide insights and prospects for the controllable preparation and performance optimization of macro CNTFs with high tensile strength in the future.

## 2. The Structure and Preparation of Carbon Nanotubes

### 2.1. The Structure of Carbon Nanotubes

**Figure 2:**
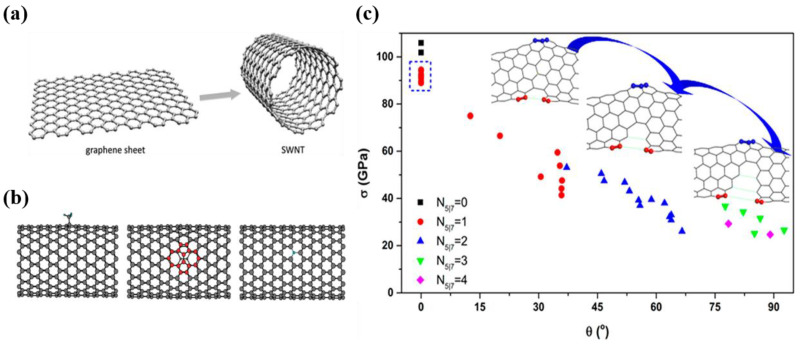
(**a**) Wrapping of graphene sheet to form an SWCNT. Reproduced with permission from [25]. Copyright 2011, Royal Soc Chemistry. (**b**) Defects on the SWCNT, from left to right: functionalization defect, SW defect, and vacancy, respectively. Reproduced with permission from [14]. Copyright 2007, Iop Publishing Ltd. (**c**) The tensile strength of carbon nanotube varies with different numbers of 5|7 defects and different chiral angles. Reproduced with permission from [12]. Copyright 2016, Amer Chemical Soc.

CNTs can be seen as a graphene sheet curled into a cylinder with a nanoscale diameter [24,25]. Figure 2a shows the structure of single-walled carbon nanotubes (SWCNTs). Carbon atoms in CNTs are linked by sp^2^ hybrid covalent bonds, which is one of the strongest chemical bonds in nature [2,3], providing graphite materials with extremely high in-plane Young’s modulus and tensile strength. The special tubular full-atomic-surface (FAS) structure composed of carbon hexagons avoids in-plane hanging bonds, folds, and concentrated local stresses in the tube wall. As a result, CNTs can exhibit excellent mechanical properties far beyond other materials (tensile strength ~120 GPa, Young’s modulus ~1.1 TPa, elongation at break ~16%, toughness ~8 GJ/m^3^) [8,26,27,28,29]. Similar to other engineering mechanics materials, the existence of defects will destroy the structural perfection of CNTs and affect the mechanical properties. The negative effect is even more pronounced for CNTs. For instance, a single vacancy defect could reduce the tensile strength of CNTs by 26% [30], and a single topological defect could lower that by 50% [12]. Despite that, the formation of topological defects is often accompanied by a high energy barrier, which can effectively protect the sp^2^ structure of CNTs. Ding et al. found that the formation energy of the five-membered and seven-membered ring pairs of topological defects was as high as 4.4 eV [31], which could effectively protect the sp^2^ structure of CNTs. Such topological protection is an important reason why CNTs are less prone to defects than other materials, such as steel or concrete.

### 2.2. Controllable Preparation of Carbon Nanotubes

**Figure 3:**
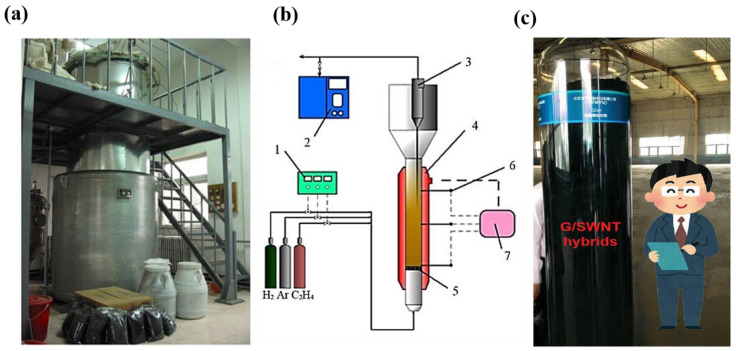
(**a**,**b**) Schematic illustration of the large-scale fabrication of CNTs by a fluidized-bed reactor. Reproduced with permission from [41]. Copyright 2008, Elsevier. (**c**) The photo of collected CNTs and graphene hybrids in mass production. Reproduced with permission from [45]. Copyright 2021, Wiley-Vch Verlag Gmbh.

Arc discharge [24,32], laser evaporation [33], and chemical vapor deposition (CVD) [34] are the three main methods for preparing CNTs. Compared with the former two methods, the CVD method has the advantages of low temperature, low energy input, and easy control of parameters and is the primary method in academic research and industrial production. The growth of CNTs by the CVD method can be divided into three stages. (i) The catalyst is in a molten state at a high temperature. (ii) The cracked carbon atoms dissolve on the catalyst, precipitate after supersaturation, and (iii) self-assemble to form CNTs [35]. There are many alternative carbon sources for the preparation of CNTs by the CVD method, such as methane, carbon monoxide, ethylene, acetylene, and ethanol. The type of carbon source has a great influence on the structure and quality of the as-grown CNTs. For example, considering the thermal cracking conditions, we thought that methane is the most appropriate carbon source for producing ultralong CNTs with perfect structure [9,36]. The catalyst is another key factor in regulating the structure and quality of CNTs. The catalysts used for producing CNTs are mainly metal catalysts, including magnetic metal catalysts (iron, cobalt, nickel), noble metal catalysts (copper, gold, silver, platinum), as well as molybdenum and tungsten, etc. Iron-based catalysts are the most commonly used and there are many reports on the preparation of CNTs with ferric chloride or ferrocene as catalysts. Ding et al. [31] also reported the role of iron nanoparticles in defect repair, explaining the high efficiency of iron-based catalysts towards CNT growth from the mechanism level. The macroscopic assembly of CNTs requires a large number of CNTs. Therefore, the large-scale production of CNTs, which can be achieved by the CVD method with relatively low cost and good controllability, is the basis of their subsequent assembly into a fiber structure. Early in 1993, Santiesteban et al. [37] first reported the fabrication of CNTs by the CVD method. The fabrication process of high-purity CNTs in large quantities based on CVD developed rapidly in the first decade [38,39]. Our group combined the traditional chemical fluidized-bed technology with the CVD method to realize the large-scale production of CNTs [40,41,42,43], which dramatically reduced the cost of CNTs (Figure 3). In addition, ultralong CNTs with macro-scale length can be synthesized by carefully regulating the growth kinetics. These ultralong CNTs possess perfect structure without any defects and can be produced at a wafer scale [9,44]. At the same time, these defect-free CNTs provide an ideal system for analyzing mechanical materials and are expected to yield new results in some branches of solid mechanics. Considering the research paradigm of the bottom-up assembly of CNTs into macrostructures as practical engineering materials, individual CNTs are the most basic structural unit. Obviously, if a large number of CNTs with defect-free or defectless structures can be synthesized and assembled into CNTFs, the excellent intrinsic properties of CNTs can be fully exploited. As a result, developing next-generation high-performance engineering materials can be promoted significantly.

## 3. Assembly Technology of Carbon Nanotube Fibers at Different Scales

The CNTFs consist of basic, tightly assembled CNT units. According to the scale of the assembled structure, we divided CNTFs into two categories. One is nanoscale carbon nanotube fibers (nanoscale CNTFs), composed of a small number of CNTs with a diameter up to hundreds of nanometers. The other is microscale carbon nanotube fibers (microscale CNTFs). The number of CNTs assembled can reach hundreds of millions with a micron-scale diameter, far more than the nanoscale CNTFs. It is a prerequisite to develop assembly technologies to combine single CNTs into nanoscale CNTFs or microscale CNTFs and maintain relatively excellent mechanical properties.

### 3.1. Preparation of Nanoscale Carbon Nanotube Fibers

**Figure 4:**
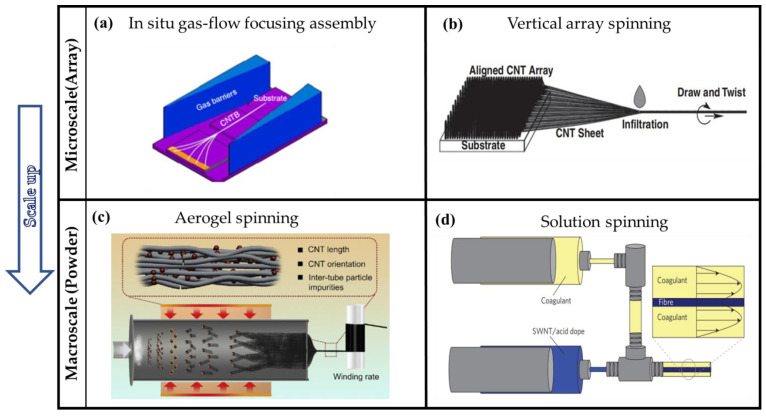
(**a**) Schematic illustration of the in situ fabrication of nanoscale CNTFs by the GFF method. Reproduced with permission from [10]. Copyright 2018, Springer Nature. (**b**) Schematic diagram of vertical-array spinning. Reproduced with permission from [51]. Copyright 2011, Pergamon-Elsevier Science Ltd. (**c**) Schematic diagram of the continuous synthesis of microscale CNTFs by FCCVD. Reproduced with permission from [18]. Copyright 2021, Elsevier Sci Ltd. (**d**) Schematic illustration of solution spinning. Reproduced with permission from [52]. Copyright 2009, Springer Nature.

Carbon nanotube bundles (CNTBs) are typical nanoscale CNTFs. Assembling a small number of CNTs into bundles requires precise nanoscale manipulation. In the early stages, researchers found that bundles with diameters of tens of nanometers can be obtained by controlling conditions using the arc-discharge method. Salvetat et al. [46] prepared SWCNT crystalline ropes by the arc-discharge method. The mean diameter of their nanotubes is about 1.4 nm, and the diameter of the bundles ranges from 3 to 20 nm with a length of several microns. The tubes were arranged in a closely-packed lattice. Espinosa et al. [47] also fabricated bundles using the arc-discharge method. The preparation of nanoscale CNTFs is a process of assembling a small number of CNTs in situ, which can be considered a bottom-up method. In contrast, to obtain CNTBs, it is also effective to assemble CNTs into a macrostructure and cut them into nanoscale CNTFs. Such segmentation from a microscale structure into the nanoscale can be considered an up-down method. Typically, Yu et al. [48] first fabricated SWCNT “paper” by the laser ablation method, and then they tore the resulting “paper” apart, which caused individual SWCNT bundles to project from the torn edge. The diameter of these nanoscale CNTFs is less than 50 nm. Based on the up-down method, Espinosa et al. [49,50] also obtained CNT bundles with diameters less than 30 nm from DWCNT mats using a mechanical exfoliation technique. From the analysis of the characteristics of these two kinds of processes, the in situ preparation of nanoscale CNTFs without post-treatments has more advantages, as the cutting strategy requires nano-precision mechanical manipulation. More structural defects or impurities will be introduced after mechanical separation, which is not conducive to the subsequent performance research. However, on the other hand, the bottom-up preparation of nanoscale CNTFs by arc discharge has its own limitations, such as high equipment requirements, low controllability, and difficult separation and purification. Therefore, it is necessary to develop a more suitable nanoscale CNTF preparation process. Our group proposed an in situ gas-flow-focusing (GFF) strategy to assemble individual tubes into bundles [10] based on the bottom-up method. As shown in Figure 4a, under the effect of the gas flow, several ultra-long CNTs gradually move towards the center and are assembled by the impact of van der Waals force. The prerequisite for preparing these centimeter-long bundles with a nanoscale diameter is that ultralong CNTs are grown based on a “kite mechanism”. Compared with the nanoscale CNTFs directly prepared by the arc-discharge or mechanical stripping method, the core of such an in situ process is the preparation of ultralong CNTs by CVD, so it has the advantages of high controllability, relatively low equipment requirements, and generally perfect fiber structure.

### 3.2. Preparation of Microscale Carbon Nanotube Fibers

The spinning techniques for microscale CNTFs are closely related to the traditional process, which can be divided into two main categories, wet spinning [16] and dry spinning [19,22,53]. Solution spinning [54] is the typical wet-spinning method, while vertical-array spinning [55] and CNT aerogel spinning [56] are the two mainstream methods of dry spinning. Understanding the characteristics of different preparation techniques is the basis for better mechanical property optimization.

#### 3.2.1. Solution Spinning

Solution spinning (wet spinning) is a relatively traditional and mature technique that has been widely used to prepare Kevlar and PAN fibers [57,58]. In the past few years, it has also been used to prepare microscale CNTFs or CNTF composites [59,60]. As Figure 4d shows, in the process of solution spinning, the CNT powder is fully dispersed in the solution with the help of a surfactant or superacid as the dispersant. The dispersed solution is injected into the coagulation bath, the solvent is dissolved in the coagulation solution, and the aggregate is precipitated to obtain continuous microscale CNTFs. The most important step for solution spinning is obtaining a highly dispersed and homogeneous CNT dispersion. Until now, a variety of effective solution systems have been developed. In 2000, Vigolo et al. [16] first produced continuous CNTFs with a diameter ranging from a few micrometers to 100 μm through this traditional spinning method. They used sodium dodecyl sulfate (SDS) as a dispersive solvent to obtain homogeneous suspensions at a high SDS concentration. The suspensions were then injected into polyvinyl alcohol using a syringe to produce CNTFs. Although the process was less efficient then, it was the beginning of the preparation of microscale CNTFs. Windle et al. [61] injected ether into the dispersion of CNTs and ethylene glycol, causing the ethylene glycol, ether, and the grown fibers to penetrate each other. Then, the ethylene glycol was heated to remove it, so that the neatly arranged microscale CNTFs were obtained with diameters ranging from 10 to 80 μm. However, such a surfactant–solvent dispersion system makes it difficult to obtain high-concentration and homogeneous CNT dispersions because of the strong van der Waals interaction between tubes. Hence, there is a need to develop more efficient decentralized systems [54]. The DuPont Company has reported the preparation technique of Kevlar fibers by dissolving polymers with concentrated sulfuric acid [57,62]. It has gradually become one of the most efficient systems in the preparation of CNTFs by solution spinning. Smalley and Pasquali et al. have developed superacid dispersion systems in the past two decades [52,54,63,64,65,66,67]. The main mechanism of using superacids to disperse CNTs is to protonate the tube wall so as to achieve efficient dispersion through electrostatic interaction. Smalley et al. dispersed CNTs in fuming sulfuric acid to obtain a CNT dispersion [54]. Pasquali et al. found that CNTs dissolved in chlorosulfonic acid could form a true thermodynamic solution in a liquid crystal phase [52,63]. The liquid crystal phase dispersion used in wet spinning can improve the spinning efficiency and obtain CNTFs with higher orientation. Their mechanical and electrical properties have been greatly improved [64,65]. However, using superacids is not friendly to the environment or equipment. Considering this problem, Pasquali et al. recently proposed a more moderate acid dispersion system. They have proposed a low-corrosive acid solvent system by using methanesulfonic acid or p-toluenesulfonic acid to form a CNT dispersion at concentrations as high as 10 g/L [68]. This system can obtain continuous and high-performance fibers, and it has higher equipment compatibility, which is conducive to mass preparation. In conclusion, the preparation of CNTFs by wet spinning has many mature technical characteristics of other fibers prepared by wet spinning, so it has more advantages in technical reliability, equipment compatibility, and further large-scale production.

#### 3.2.2. Vertical-Array Spinning

As shown in Figure 4b, vertical-array spinning means continuously extracting CNTs connected by van der Waals action from spinnable vertical array CNTs (similar to cocoon spinning) to prepare macroscopic fibers. Vertical-array spinning has strict requirements on the structure of CNT arrays. In order to draw out CNTFs continuously, the vertical arrays of CNTs should be high in height and density while the orientation should be very uniform [69]. In 2002, Fan et al. [55] extracted CNT yarns from a 100 μm-high super-aligned CNT array with tweezers. Superaligned CNT arrays are the ideal raw materials for vertical-array spinning. The prepared continuous microscale CNTFs were 30 cm in length and 200 μm wide. Baughman et al. [70,71] first prepared CNT yarns with stable torque by twisting the yarns drawn out from the vertical arrays. The drawn yarns are very sticky due to their clean surface and extremely high specific surface area. They stick to the surfaces once they touch other objects and cannot be taken off again. This greatly inhibits the development of practical applications for CNT yarns. To solve this problem, Fan et al. [69] designed a new array-spinning process. After they had drawn the yarns out of the super-aligned array, the yarns were pulled through the ethanol droplets, and the centimeters-wide yarns shrank into a fiber structure of 20–30 μm in diameter. In this way, the processed fibers are more convenient for transfer or post-processing, thus making device applications possible. As mentioned above, twisting and solvent contraction are important steps in array spinning. These two improved techniques not only make it easier to produce fibers by array spinning but also can significantly improve the tensile strength and other properties. Li et al. [51] applied the steps of twisting and ethanol infiltration to controllably fabricate continuous microscale CNTFs using a spinning machine (Figure 4b). Compared with the solution, the biggest advantage of array spinning is that there is no need to prepare a high-concentration and homogeneous dispersion, which means that the structural damage of the CNTs can be reduced, and the extraordinary properties of CNTs can be fully exploited. However, the vertical-array spinning technique is relatively immature, it is difficult to achieve industrial scale-up, and the production cost is high. Therefore, this technique is suitable for the fabrication of small multifunctional devices that require microscale CNTFs.

#### 3.2.3. Aerogel Spinning

Aerogel spinning is a continuous spinning process involving three phases. The process is shown in Figure 4c. The CNTs for aerogel spinning are generated by floating catalytic chemical vapor deposition (FCCVD). The catalyst (generally ferrocene) is firstly fed into a high-temperature reactor and reduced by hydrogen. Then, a carbon source such as methane or ethanol is cracked on the catalyst to form a tubular fiber precursor structure. Under gas flow, the precursor is passed through water or other coagulation solution to achieve rapid injection and fibrosis due to the capillary fineness of the liquid. According to the characteristics of the process, it can be considered that the precursor of microscale CNTFs, similar to powder, is in situ assembled into a macro fiber structure. Initially, only the gas–solid phase was involved in aerogel spinning. In 1998, Cheng et al. [38,72] obtained web-like, silver-black, light, and thin materials made of large quantities of bundles ranging from 10 to 40 nm in diameter, mainly SWCNTs. This was the beginning of the direct aerogel spinning of microscale CNTFs by FCCVD. Later, Zhu et al. [73] prepared 20 cm-long CNT yarns with a diameter of 0.3 mm using a similar method. However, the yarns they prepared were isolated and the diameter of the same yarn was not uniform. To collect a large number of continuous fibers, Windle et al. [56] designed different rotating spindles to wrap the aerogel. By drawing CNT aerogel directly from the hot reaction zone, they found that continuous fibers could be collected without length limitations. Similar to vertical-array spinning, aerogel spinning is also improved by solvent shrinkage. Windle et al. [17] densified the fibers by using acetone vapor. Li et al. [74] developed the water-sealing technique to draw fiber precursors into a spindle in water and then collected them in a spindle in the air. Through such a process, yarns with a length of several kilometers can be obtained, and their quality is close to conventional textile yarns. Like vertical-array spinning, aerogel spinning does not need the dispersion step. That means the fibers obtained by this spinning process can also take full advantage of the excellent mechanical properties of CNTs. In addition, wet spinning is based on the traditional spinning process and the technology is relatively mature and easy to achieve industrialization [22,75,76]. The direct spinning method of aerogel has also been capable of large-scale preparation. Li et al. reported that they could fabricate kilometer-level CNTFs with high tensile strength by FCCVD [18]. Nanocomp, an American company, can fabricate 10 km-long CNT spinning threads and has promoted their practical application in the aerospace industry [77,78]. Therefore, the aerogel direct spinning method based on FCCVD is a very ideal system for studying mechanical property transfer across scales and developing practical applications based on CNTs.

Figure 4 shows the schematic diagram of different assembly processes for CNTFs at different scales. The process of in situ gas-flow focusing assembly or continuously drawing fibers from vertical CNT arrays are precise assembly techniques with nanoscale structure control. Although these microscale assembly techniques have advantages in structure and property control, it is difficult to achieve large-scale preparation, which means a lack of suitable application scenarios. In contrast, solution spinning and aerogel spinning as typical macroscale assembly techniques have more potential for industrial production. Despite that, there are still many significant problems to be solved for the excellent tensile strength transfer to a larger scale. From a bottom-up perspective, it may be effective to apply the methods of fabrication and property optimization of microscale materials to macroscale materials.

## 4. Tensile Property of Carbon Nanotube Fibers at Different Scales

### 4.1. Tensile Strength of Single Carbon Nanotubes

A single CNT is a basic structural unit of CNTFs; the mechanical properties determine the macro properties. Tensile strength is a crucial index for evaluating the mechanical properties of materials. For nanoscale single CNTs, precise experiments for tensile strength measurements require delicate nanomanipulation techniques. The theoretical model based on quantum mechanics calculation showed that the tensile strength of a single CNT could be as high as 120 GPa [30]. Several possible defects (SW defects, vacancies, doped atoms, etc. [30,79,80,81,82]) significantly influence the mechanical properties of CNTs [83,84]. Many theoretical studies have revealed the excellent intrinsic tensile strength of CNTs and predicted that the presence of defects would greatly reduce the tensile strength, which has been discussed in the former structure section. The tensile strength of CNTs could be reduced by orders of magnitude due to the emergence of a small number of topological defects [12,85]. Experimental studies confirmed these characteristics of the tensile strength of single CNTs. Early research on the mechanical properties of CNTs mainly relied on atomic force microscopy [27,86] or electron microscopy [3,26,28,87,88], and few experiments could observe a single CNT with an ultra-high tensile strength of more than 100 GPa. Using in situ scanning electron microscopy, Yu et al. obtained the tensile strength of multi-walled carbon nanotubes, ranging from 11 to 63 GPa, and the outer diameters ranged from 13 to 36 nm [28]. Chen et al. tested the tensile strength of double-walled carbon nanotubes and triple-walled carbon nanotubes (TWCNTs) with diameters ranging from 1.8 to 3.0 nm and obtained strengths ranging from 13 to 46 GPa [11]. They considered that the poor results were caused by defects in the CNTs. Takakura et al. investigated the tensile strength of SWCNTs with diameters ranging from 1.5 to 3.0 nm. The tensile strength was in the 25~66 GPa range, showing a decreasing trend with the increase in diameter [89]. With the development of controllable preparation technology for CNTs, the length and properties of CNTs have been significantly improved. Espinosa et al. fabricated MWCNTs with a diameter of 15.71 nm and measured a tensile strength of 110 GPa [90]. Zhang et al. fabricated ultra-long CNTs with a length of 55 cm, and their tensile strength was up to 120 GPa [9]. Bai et al. also fabricated centimeter-long and defect-free CNTs with a tensile strength of 118.9 ± 4.5 GPa [15]. Therefore, a single CNT with tensile strength very close to the theoretical limit can be obtained through experiments. From the bottom-up perspective, defect-free single CNTs, as a structural unit of a macrostructure, are a crucial carrier for the study of property transfer across scales. The controllable preparation of defect-free or defectless CNTs is the basis for the assembled CNTFs with high tensile strength.

### 4.2. Tensile Strength of Carbon Nanotube Fibers at Different Scales

Although single CNTs have shown excellent tensile properties in both theoretical and experimental studies [15,30], it is rather tough to transfer the remarkable properties of single CNTs to larger CNTs across the scale. According to the characteristics of the preparation process, the bundle with tens of nanometers in diameter and the fiber with hundreds of microns can be regarded as the bottom-up assembly of a single CNT. However, the tensile strength decreases significantly when the CNTs are clustered to form macro fibers, ropes, or even bundles.

#### 4.2.1. Nanoscale Carbon Nanotube Fibers

**Figure 5:**
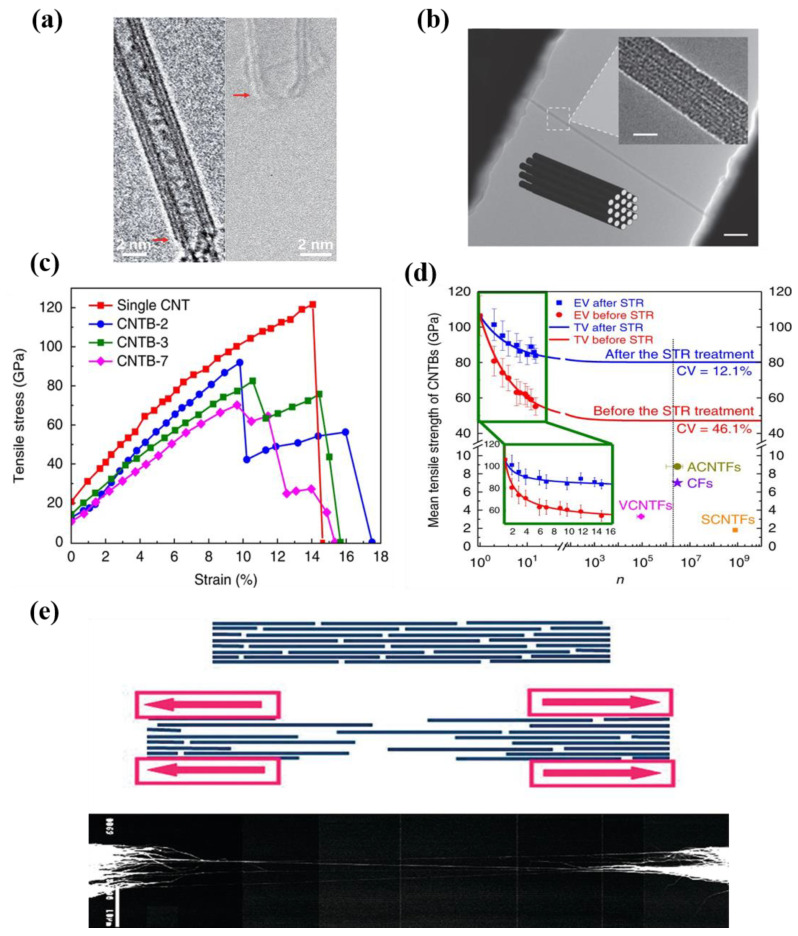
(**a**) TEM images of the flat fracture surfaces (marked by arrows) of the CNTs. Left: static tensile fracture surface; right: fracture surface after cyclic loading. Reproduced with permission from [15]. Copyright 2020, Amer Assoc Advancement Science. (**b**) TEM top view images of a DWCNT bundle (Scale bar: 200 nm). Insert: High-resolution TEM image of the suspended DWCNT bundle (Scale bar: 20 nm). Reproduced with permission from [50]. Copyright 2011, Wiley-Vch Verlag GmbH. (**c**) Stress–strain curves for single CNTs and CNTBs. (**d**) The relationship between the mean tensile strength of CNTBs and their component number before and after STR treatment. (**c**,**d**) Reproduced with permission from [10]. Copyright 2018, Springer Nature. (**e**) Top: Model of CNTFs assembled from individual CNT units. Middle: Model of tensile fracture of CNTFs. Bottom: SEM micrograph of the tensile fracture of actual CNTFs (Scale bar: 10 μm) Reproduced with permission from [91]. Copyright 2011, Amer Chemical Soc.

For nanoscale CNTFs such as bundles or yarns, the number of assembled CNTs is far less than that of microscale CNTFs. The bundle formed by the aggregation of several or dozens of CNTs is a crucial bridge from the nanoscale CNTFs to the microscale ones. Based on the controllable preparation of ultra-long CNTs, the author’s group fabricated nanoscale CNTFs that are centimeters long with tensile strength close to that of single CNTs [10]. As shown in Figure 4a, CNTBs consisted of only 2–15 CNTs with diameters between 2 and 15 nm. The tensile strength of such CNTBs could be up to 80 GPa, which were the only nanoscale CNTFs with tensile strength close to that of a single CNT at present. Espinosa et al. used an in situ transmission electron microscope to test DWCNT bundles with diameters ranging from 10 to 30 nm, as shown in Figure 5b [50]. They obtained a tensile strength of 17 GPa and a tensile modulus of 0.7 TPa. Cheng et al. prepared SWCNT strands with a diameter of 10–40 nm [38]. Although the diameter of the bundle was very small, the process was developed at a relatively low maturity for fiber assembly. The tensile strength was only 3.6 ± 0.4 GPa in the test [72]. Yu et al. fabricated SWCNT bundles with diameters ranging from 19 to 41 nm and studied their tensile fracture behavior using scanning electron microscopy and atomic force microscopy [48]. The tensile strength of SWCNT bundles ranged from 13 to 52 GPa (mean 30 GPa). The problem of the cross-scale transfer of tensile strength is not solved for most nanoscale CNTFs, though the diameters of these fibers are all less than 100 nm. Nanoscale CNTFs with smaller diameters are more promising to be higher in tensile strength, which should be attributed to their fewer defects and well-controlled tube–tube interactions. Since the diameters of nanoscale CNTFs are lower than 100 nm, the control of defects and tube–tube interactions can still be effective. The tensile strength of nanoscale CNTFs can easily exceed the order of 10 GPa, however, it will be very difficult for microscale CNTFs to maintain such a high level of strength.

#### 4.2.2. Microscale Carbon Nanotube Fibers

For microscale CNTFs, the number of CNTs assembled can reach hundreds of millions, far more than the CNTBs. Since the fiber has a micron diameter, the microscale CNTFs can be considered as a bottom-up assembly of nanoscale CNTFs. Vigolo et al. [16] obtained CNTFs with a tensile strength of only 0.15 MPa. The fiber diameter can be distributed in the range of several microns to 100 microns. Smalley et al. [54] obtained CNTFs with a diameter of less than 1 micron and the tensile modulus and tensile strength reached 120 GPa and 116 MPa, respectively. Pasquali et al. [64] prepared CNTFs with diameters of approximately 9 μm and tensile strength of about 1.3 GPa. Later, they improved the tensile strength of microscale CNTFs to 4.2 GPa after process optimization [65]. Zhu et al. obtained microscale CNTFs with an average diameter of 5 μm and tensile strength up to 3.3 GPa [92]. Windle et al. optimized the post-treatment process and prepared microscale CNTFs of less than 20 μm in diameter with a tensile strength of 9 GPa [17]. Similar to Windle et al., Wang et al. [5] fabricated microscale CNTFs with diameters between 5 and 9 μm and tensile strength ranging from 3.76 to 5.53 GPa. Further, they prepared CNT films with tensile strength of 9.6 GPa [4]. The changes in the tensile strength of microscale CNTFs from a low to a high level are closely related to the scale of fibers and the maturity of the assembly technology. Due to the countless number of CNT units in microscale CNTFs, the efficiency of controlling the defects and tube–tube interactions will be significantly decreased, resulting in a relatively low tensile strength. As shown in Table 1, the tensile strength of CNTs at different scales is listed. The tensile strength of single CNTs and CNTFs with nanoscale diameters has been able to approach the theoretical level. Although significant progress has been made in the controllable preparation and process optimization of microscale CNTFs, their tensile strength is significantly inferior to that of single CNTs and nanoscale CNTFs, which is the typical undesirable performance transfer across different scales.

### 4.3. Characteristics of Tensile Strength Transfer across Scales

The bottom-up composition of CNTs is similar to that of carbon fiber and cable-stayed bridge wires. Weibull distribution and the Daniel effect can describe the strength distribution of single filaments and carbon fibers [93,94,95], which have been used to describe the ideal state of performance transfer when CNTs are assembled into macroscopic fibers. Daniel et al. found that the bundles consisting of a large number of monomers with a tensile strength obey the Weibull distribution [96,97,98,99], and the average tensile strength E_σ_(n) is:

$$E_{\sigma }\left(n\right){=\sigma }_{0}\left[1-F\left(\sigma_{0}\right)\right]{+c}_{n}/n$$

where F(σ) is the breaking probability of a single CNT under stress ≤ σ. Cn is a variable related to the number of components (n). Based on the Weibull distribution and the Daniel effect, Bai et al. proposed a mathematical model to describe the relationship between the tensile strength of nanoscale CNTFs and their component numbers and initial strains [10]. As shown in Figure 5d, the experimental results fit well with the theoretical calculation. With increasing *n*, there is a quasi-exponential decrease in the mean tensile strength of the nanoscale CNTFs. The mathematical model results also showed that the tensile strength reaches a constant value for a number of constituent tubes larger than a certain value, which is very similar to the characteristics of steel wire in cable-stayed bridges [98]. However, the current prepared microscale CNTFs could not confirm this theoretical prediction, as the maximum tensile strength of microscale CNTFs with macroscopic structure was less than 10 GPa, which was much lower than the tensile strength of single CNTs and nanoscale CNTFs. Analyzing the reasons for this phenomenon is of great significance for the rational design of performance optimization strategies in the furture. The sp^2^ hybridized C-C covalent bonds and fewer defects are the structural basis for the ultrahigh tensile strength of CNTs [9,26,100]. As Figure 5a,b show, individual CNTs with perfect structure and well-aligned bundles can make full use of C-C covalent bonds. However, the van der Waals forces are more crucial to be considered when scaling up from the nanoscale to the microscale. For example, the slip between tubes caused by weak tube–tube interactions is an important reason for the low tensile properties of macroscopic fibers. Windle et al. [91] proposed a model of the assembly of CNTFs and their tensile fracture (Figure 5e). Microscale CNTFs are composed of a large number of short CNTs, which means that tube–tube slip asynchronously is more likely to happen before the short CNTs fracture. In other words, the tensile properties of microscale CNTFs depend largely on van der Waals forces rather than C-C covalent bonds. This may be a reason for the unsatisfactory transfer of mechanical properties across scales of CNTs. Therefore, optimizing tube–tube interactions is fundamental as defect control to improve the tensile strength of CNTFs at different scales. Suppose that the macrostructure is composed of numerous ultra-long, defect-free, or defectless CNTs with continuous length, perfect structure, uniform orientation, and uniform initial stress distribution, then there will be a high possibility that its tensile strength can still maintain a rather high level.

## 5. Optimization Strategies for the Tensile Strength of Carbon Nanotube Fibers at Different Scales

### 5.1. Defects Control

Defects have a fatal negative effect on the intrinsic properties of CNTs [12,79,101]. In a large number of early experimental studies on the tensile strength of single CNTs, the measured tensile strength was significantly lower than 50 GPa due to the presence of the many structural defects of the CNTs [11,28]. Zhang et al. [9] and Bai et al. [10] fabricated defect-free CNTs with tensile strength close to the theoretical value. Further, Bai et al. assembled these defect-free CNTs into a bundle structure and the tensile strength reached up to 80 GPa, which was also close to the theoretical level. The defect-free structure enabled the desired transfer of tensile strength from single CNTs to nanoscale CNTFs. Due to the bottom-up characteristics of the assembly of CNTFs, the large-scale production of CNTs with fewer defects is the basis of high-strength microscale CNTFs. In the early process of fiber preparation by wet spinning, the raw powder of CNTs had many defects, and the maximum tensile strength of the fibers was less than 1 GPa [16,52]. During the development of wet spinning, Pasquali et al. continuously improved the tensile strength of the fibers, reaching a maximum of 4.2 GPa [65]. This is mainly attributed to the progress in preparing defectless CNTs as raw materials to produce fibers. Recently, Kim et al. have continuously synthesized CNTs with high crystallinity (IG/ID > 60), high aspect ratio (>17,000), and high yield (>6 mg/min) [103]. The high Raman IG/ID signal implied the good quality of the prepared CNTs. The specific strength and modulus of the microscale CNTFs prepared using the products were 2.94 N tex^−1^ and 231 N tex^−1^, equivalent to the best carbon fibers. This further proves that it is essential to obtain perfect and defect-free CNT raw materials in order to prepare CNT macrostructures with excellent properties.

In addition to the defects introduced by CNTs themselves, the bottom-up assembly process of single tubes into a macroscopic structure also inevitably introduces many defects. More defects will be introduced if vigorous treatments such as superacid and ultrasonic-assisted dispersion are applied [14]. As a result, this will fundamentally reduce the tensile strength of the microscale CNTFs. It is essential to reduce the defects introduced by the assembly process. Smalley et al. [54] prepared CNTFs with very low mechanical strength due to the use of fuming sulfuric acid in the dispersion process, which can easily damage the structure of CNTs and introduce more defects. Pasquali et al. [64] reduced the defects by a liquid crystalline phase system, thus significantly improving the tensile strength of the fibers [52]. The number of defects will be reduced dramatically during dry spinning as it does not require dispersion, a process that inevitably damages the structure. During the vertical-array spinning process, the materials are highly purified vertical CNT arrays, which means that fewer defects will be introduced from both the spinning process and the raw materials themselves. The maximum tensile strength of CNTFs with few defects has reached 3.3 GPa [92]. As a one-step process, aerogel array spinning can assemble CNTs into fibers in situ. Obviously, this process also significantly reduces the introduction of structural defects. Li et al. optimized the process conditions and produced a kilometer-level CNTF with a tensile strength of 3.5 GPa without post-treatment. Dry spinning is a cleaner system that reduces the number of defects in the CNT macrostructure. The tensile strength of CNTFs prepared by dry spinning can be up to 10 GPa after post-treatment [4,17], which is generally higher than that of CNTFs with more structural defects prepared by wet spinning. The difference in defect control caused by different technological processes is a key factor to optimizing the tensile strength.

### 5.2. Control of Tube–Tube Interactions

Improving the process to achieve good cross-scale transfer is a great challenge. Windle et al. pointed out that the tensile strength of CNTFs depends on the interactions between the tubes [91]. Many factors directly or indirectly affect the interactions between tubes and then affect the tensile strength of the assembled macro fibers. Understanding and controlling the tube–tube interactions are significant.

#### 5.2.1. Initial Strain

**Figure 6:**
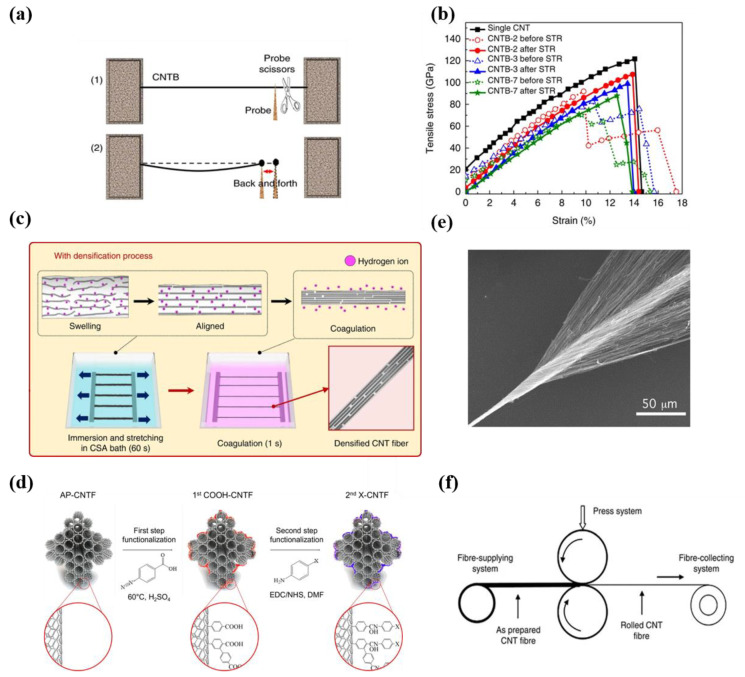
(**a**) Schematic diagram to make the initial stress of each tube more uniform. (**b**) Stress–strain curves for a single CNT and for CNTBs before and after the stress uniformity treatment. (**a**,**b**) Reproduced with permission from [10]. Copyright 2018, Springer Nature. (**c**) Schematic of the stretching and densification of CNTFs. Reproduced with permission from [6]. Copyright 2019, Springer Nature. (**d**) Schematic of the two-step chemical molecule cross-linking. Reproduced with permission from [120]. Copyright 2021, Elsevier Sci Ltd. (**e**) SEM images of a twisted CNT yarn. Reproduced with permission from [70]. Copyright 2004, Amer Assoc Advancement Science. (**f**) A schematic of the system for rolling CNTFs. Reproduced with permission from [5]. Copyright 2014, Springer Nature.

The bottom-up assembly of CNTs is similar to the steel wire of a cable-stayed bridge. The main cable of a cable-stayed bridge comprises tens of thousands of untwisted steel wire bundles with a tensile strength of about 2 GPa and a diameter of several millimeters [102,103,104]. During construction, the steel wire bundles should be divided into hundreds of groups, and the initial strain needs to be as uniform as possible to ensure uniform force. When CNTs are assembled into bundles or fibers from bottom to top, the initial tension of each tube is inevitably different. According to the Daniel effect, the tensile strength of bundle and fiber will decrease rapidly with the increase in the number of tubes if the initial strain is not uniform [96]. Bai et al. studied the initial strain of CNTs in nanoscale CNTFs. The measured tensile strength is shown in Figure 6b. The tensile strength of CNTBs decreases with the increasing number of components, and the breaking process under tensile loading exhibits a multi-stage, one-by-one process. This indicates that the initial stress distribution of CNTs in the nanoscale CNTFs is not uniform, so the CNTs in the fibers cannot bear the load synchronously and equally, and the components break one by one, which leads to the decline of the overall tensile strength. This is an essential point of view elucidating the difficulties during performance transfer across scales. Based on this analysis, the research group proposed a synchronous tightening and relaxing (STR) strategy, as shown in Figure 6a,b, in which the initial stress of the CNTs in the fibers is released to a narrow distribution through nano-manipulation, thus increasing the tensile strength of the nanoscale CNTFs from 47 GPa to more than 80 GPa [10]. Therefore, the tube–tube interaction is an important guarantee of excellent tensile performance, and STR is an effective strategy to make the initial stress of CNT components uniform and improve the tensile strength across scales. Recently, Kim et al. [6] developed a rapid and continuous method to produce highly aligned and densified CNTFs. As Figure 6c shows, the fibers obtained by direct aerogel spinning were dissolved in chlorosulfonic acid, and the alignment of CNTs along the axial was improved after stretching. Such an improvement also made the initial strain of CNTs in microscale CNTFs more uniform. As a result, the tensile strength of CNTFs was increased from 2.1 N tex^−1^ to 4.44 N tex^−1^, indicating the significance of the uniform initial stress.

#### 5.2.2. Length-Dependent Interactions

CNTs are the basic structural units of CNTFs from bottom to top. When the length of the CNT component increases, the contact area between adjacent CNTs also increases, which improves the local transfer efficiency between tubes and enhances the mechanical properties of fibers. Fibers composed of longer CNTs mean that there are fewer ends. In that case, the force on each part will be more uniform, and the tensile strength will be improved [105,106]. Therefore, increasing the length of CNT units in the macrostructure is an effective strengthening strategy. Pasquali et al. systematically studied the influence of CNT structure on the tensile performance of microscale CNTFs prepared by solution spinning [107]. The results showed that the tensile properties of CNTs were mainly influenced by their aspect ratio rather than other factors such as the number of tube walls, diameter, and crystallinity. In 2013, Pasquali et al. improved the spinning process and used CNTs with an average length of 5 μm as raw materials to prepare high-performance multifunctional fibers with an average tensile strength of 1 GPa [64]. Recently, Pasquali et al. dissolved CNTs with a high aspect ratio (12 μm of average length) in chlorosulphonic acid and obtained microscale CNTFs with a tensile strength of 4.2 GPa, which is far higher than the fibers spun by the CNTs with an average length of 5 μm [65]. By adjusting the growth kinetics, CNT arrays with a higher height can be fabricated, and a macrostructure composed of longer CNT units can be obtained by spinning. Zhu et al. studied the effect of array height on the tensile strength of spun fibers [108]. They fabricated 300 μm-, 500 μm-, and 650 μm-high vertical arrays and spun them into fibers. These fibers’ tensile strengths are 0.32, 0.56, and 0.85 GPa, respectively. Further, Zhu et al., fabricated fibers with longer (1 mm) individual CNTs. The tensile strength of the fabricated fibers can be up to 3.3 GPa. Windle et al. [17] and Li et al. [18] fabricated fibers based on the FCCVD method, and also proved that with the increase in single CNT length, the tensile strength of the macro structure of CNTs would be higher. The enhancement of the interaction force between tubes results from an increase in the length of constituent CNTs, which can reduce the slip between tubes and make full use of the mechanical properties of the CNTs.

#### 5.2.3. Packing Density

The increased packing density can reduce the fiber cross-sectional area, which will effectively reduce the distance between tubes and improve the tube–tube interactions so that the load transfer capacity will be increased [55,109,110]. Xie et al. fabricated an MWCNT rope with a tube spacing of about 100 nm, and the tensile strength was measured to be 4 GPa [111]. In contrast, Bai et al. assembled tubes into bundles tightly without pores, and the tensile strength of such dense nanoscale CNTFs can be improved to as high as 47 GPa without any post-treatments [10]. For microscale CNTFs, there are many effective methods to increase the packing density [112,113]. Baughman et al. introduced the twisting step in this spinning method [70], which increased the fiber density to 0.8 g/cm^3^ and the tensile strength to 150~300 MPa, as shown in Figure 6e. Li et al. [56] introduced a twisting and collecting device at the end of the furnace, resulting in the continuous in situ preparation of microscale CNTFs with a tensile strength of 3.24 GPa (1 mm pinch) [5,17]. Physical and chemical crosslinking treatment [114,115] and densification solvent [116,117] are also effective methods for fiber densification. Polymers such as polyvinyl alcohol and epoxy resin are good densifying solvents, which will contribute to enhancing the load transfer capacity and effectively improving composite fibers’ mechanical properties [118,119]. Jiang et al. densified the spun fibers with PVA solution to obtain CNT composite fibers with a tensile strength of 2 GPa [117]. Dalton et al. prepared a CNT/polyvinyl alcohol composite by wet spinning [59,60], and an ultimate tensile strength of 1.8 GPa was obtained in the test. For aerogel spinning, there are many holes in the prepared fibers. Li et al. [122] reported the densification of different solvents and found that densification using non-volatile solvents with high polarity could significantly enhance the strength. Li et al. [123] were also inspired by straw bundling and used the character of self-contraction of silk fibroin to densify CNTFs locally. The tensile strength of CNTFs was enhanced from 355 MPa to 960 MPa. Mechanical densification is a simpler and more effective post-treatment densification technique. Figure 6f shows that by collecting CNT cylinders and introducing the rolling strategy for densification, Wang et al. [6] obtained a CNT macrostructure with an average tensile strength of 4.34 GPa, which is 12 times higher than that of unrolled fibers. After rolling treatment and the optimization of the winding rate, the density of fibers was significantly improved from 0.53 g cm^−3^ to 1.85 g cm^−3^, and the tensile strength was tested as high as 9.6 GPa, which is the highest value among macrostructures of CNT until now [4]. These indicated that rolling is an effective strategy to improve the mechanical properties of microscale CNTFs. The above results have addressed the significance of higher packing density for the improvement of tensile strength.

## 6. Prospects for the Application of High-Strength Carbon Nanotube Fibers

### 6.1. Structural Reinforcing Material

**Figure 7:**
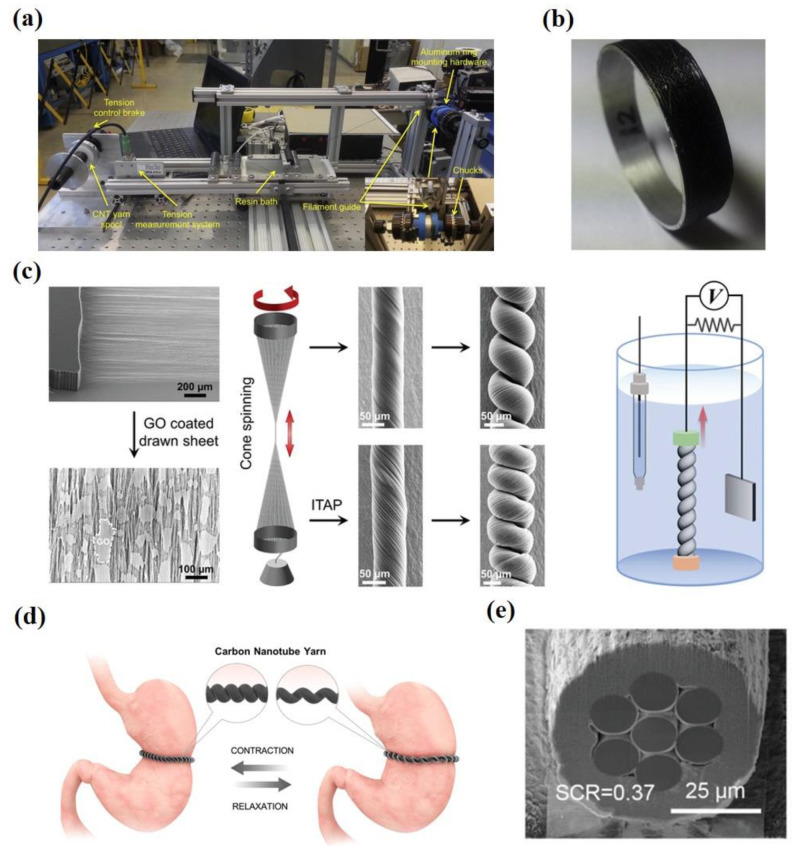
(**a**) Equipment in which Al rings are overwrapped with CNTFs. (**b**) Al ring overwrapped with Epon 828/CNT yarn. (**a**,**b**) Reproduced with permission from [123]. Copyright 2016, Elsevier Sci Ltd. (**c**) SEM images and illustration of cone spinning for fabricating twisted and coiled neat CNT yarns from forest-drawn CNT sheets and its designed application device. Reproduced with permission from [129]. Copyright 2022, Wiley-Vch Verlag Gmbh. (**d**) Coiled CNT yarns for stomach sensors. Reproduced with permission from [130]. Copyright 2019, Amer Chemical Soc. (**e**) Cross-sectional SEM images for sheath-driven PI_PDMS@CNT muscles. Reproduced with permission from [131]. Copyright 2022, Wiley-Vch Verlag Gmbh.

Due to their outstanding intrinsic mechanical properties, CNTs are ideal candidates for structural reinforcement materials. Although the tensile strength of CNTFs is far from the theoretical value, there have been many CNTFs with a tensile strength higher than T1000, one of the strongest commercial carbon fibers [4,120]. As structural reinforcement materials, CNTFs can greatly improve the strength of materials such as polymers [119,121,122], metals [123,124], and ceramics [125,126], thus playing an important role in many fields such as aerospace and building materials. NASA has identified CNTFs as an alternative reinforcement material [122]. They used continuous CNT yarns reinforced Ultem to build the frame of a quadcopter. They also demonstrated the application value of CNT yarns in coating materials (Figure 7a,b). The breaking load of a bare aluminum ring was about 5000 N while the breaking load of an aluminum ring coated with Epon 828/CNT yarn could be higher than 11,000 N [123]. With lightweight and high-strength mechanical properties, CNTFs are ideal materials for various kinds of armor, especially for body armor applications [127,128]. Lee et al. [128] studied the dynamic strengthening phenomenon of CNTFs under extreme mechanical impulses. They found that the kinetic energy absorption properties of CNTFs are superior to other high-performance fibers such as nylon, Kevlar, and aluminum monofilament. Structural reinforcement is one of the most important applications of CNTs. With the breakthrough of high-strength CNTFs, more achievements can be realized in high-end fields. Even the space elevator [1] can be possible.

### 6.2. Energy Storage

In recent years, new energy development has attracted attention to energy storage. Energy storage devices are an important emerging application field of CNTFs, and some advances have been made in device design and fabrication. CNTFs with high mechanical properties are important materials for mechanoelectrical energy conversion [21,132,133]. Baughman et al. [134] designed a CNT yarn harvester that can convert tensile or torsional mechanical energy into electrical energy. The peak electrical energy generated by the coiled fibers was 250 W/kg as the stretching cycle frequency reached 30 Hz. At mechanical frequencies between 6 and 600 Hz, the peak electrical energy generated per harvester weight was higher than that generated by any prior-art mechanical energy harvesters. However, the conversion efficiency of the twistron fibers was not high enough. Recently, Baughman and Kim et al. [129] proposed some effective strategies to improve energy conversion efficiency. As shown in Figure 7c, these improvement strategies include optimizing the orientation, applying tensile stress, electrothermal pulse annealing under stress, and loading graphene nanosheets [129,135]. The optimized twistrons reached a peak power output of 12-fold that of the mechanical energy collector in the prior art at a frequency of 30 Hz. These electromechanical conversion devices are expected to be applied in wave energy generation, using human motion to power sensors and energy storage, and other emerging fields of energy storage [130,136,137,138]. Figure 7d shows that Kim et al. [130] designed a sensor for detecting gastric deformations based on coiled CNT yarn.

### 6.3. Artificial Muscle

Many studies have reported that the prepared CNTFs possess high mechanical and electrical properties. Pasquali et al. [65] reported that fibers prepared by wet spinning had a tensile strength of 4.2 GPa and electrical conductivity of 10.9 MS∙m^−1^, comparable to copper. Artificial muscle materials need excellent mechanical and electrical properties. Therefore, the research on artificial muscle based on CNTFs has attracted much attention. Kim and Baughman et al. [139,140] fabricated artificial muscles by spinning twisted CNTs, exhibiting the contractile motion of an all-solid-state stretching muscle. Further study showed that the tension and contraction of two-ply coiled CNT yarn were as high as 16.5% under voltage driving, which was about 30 times higher than natural muscle [141]. They increased the capacitance by introducing graphene, and the tensile stroke of artificial muscle made with CNT yarns was increased two-fold [139]. Recently, they designed an artificial muscle structure with a polymer core and CNT sheath (Figure 7e). This structure can achieve an average power density of 12 kW/kg, which is 42-times that of human skeleton muscle [131]. Fibrous artificial muscles are being investigated for applications such as robotics, prostheses, and exoskeletons. The design and manufacture of related devices have been relatively mature. CNTF-based artificial muscles are also more multifunctional.

## 7. Conclusions

Carbon nanotubes are a kind of ultra-light and high-strength material. The fiber structure composed of CNTs is expected to be a significant breakthrough for the next generation of high-strength mechanical materials. However, the mechanical properties such as the tensile strength of CNTFs are still far lower than the theoretical levels. Further research on the preparation and assembly of CNTs is still urgently needed for the revolutionary development of nanomaterials. In this review, we have analyzed the controllable preparation and tensile strength of CNTs at different scales. Based on recent years’ research on the controllable preparation of CNTFs, the significance of defect control and efficient treatment process to control tube–tube interactions is emphasized towards ultra-high-strength CNTFs. We introduce the unique structure and large-scale preparation of CNTs, which is the basic unit of nanoscale assembly. The significance of defect control for high-strength CNTFs is emphasized. In addition, the mechanical strength and toughness can be significantly improved by eliminating the tube–tube non-uniform interactions or other post-treatment interactions. Based on the bottom-up method, the mass production of defect-free CNTs and accurate assembly into the macro-scale with fewer defects, fine alignment, and higher density is the priority to obtain macro-scale assemblies with excellent tensile strength. In addition to tensile strength, mechanical properties such as fatigue, bending, and torsion, as well as electrical and thermal properties, face a similar problem of performance transfer across scales. With further analysis of the influencing factors and optimization strategies for the cross-scale transfer of other properties, the excellent intrinsic properties of CNTs can be fully utilized, and CNTs can play a more significant role in many fields.

However, there are still many challenges to be solved. The first is the definition of high-end application scenarios for these super strong, super tough materials. There are ideal concepts, such as space elevators and flywheels, but they all require cross-disciplinary assistance that is not available with current technology. The second is that the continuous length of a single tube can be only meter-level, while tens of billions of tubes are needed to make a macroscopic fiber even with a kilometer in length. Such non-damage welding and inter-tube orientation of nanoscale fibers are challenges to science, engineering, and material manufacturing. Therefore, tremendous breakthroughs at the technical level are still needed. Finally, in order to further improve the strength and achieve practical application, the large-scale preparation of CNTs with defect-free or defectless structures is crucial. However, without engineering input in application scenarios, the development of the large-scale preparation of such nanomaterials will be an unspontaneous and challenging process. Despite of that, we believe that the continuous attention from researchers in the fields of emerging energy and materials can finally find solutions to those tough but significant problems, such as the preparation and performance optimization of cutting-edge fiber materials like CNTFs.

## Figures and Tables

**Table 1:** Tensile strength of CNTs at different scales.

Scales	Diameter	Carbon Nanotube	Tensile Strength (GPa)	Ref.
Single tubes	2.0 nm	DWCNTs	118.9 ± 4.5	[15]
	1.0 to 4.0 nm	SWCNTs, DWCNTs, TWCNTs	120	[9]
	15.71 nm	MWCNTs	110	[90]
	1.5 to 3.0 nm	SWCNTs	25 to 66	[89]
	1.8 to 30 nm	DWCNTs, TWCNTs	13 to 46	[11]
	13 to 36 nm	MWCNTs	11 to 63	[28]
Nanoscale CNTFs	10.0 to 25.0 nm	SWCNTs, DWCNTs, TWCNTs	47 to 80	[10]
	19 to 41 nm	SWCNTs	13 to 52	[48]
	10.8 to 27.9 nm	DWCNTs	1.5 to 17.1	[50]
	10 to 40 nm	SWCNTs	3.6 ± 0.4	[38,72]
Microscale CNTFs	/	DWCNTs	9.6	[4]
	7.0 to 20.0 μm	DWCNTs	9	[17]
	8.0 to 9.8 μm	SWCNTs	4.2	[65]
	5.0 to 9.0μm	DWCNTs	3.76 to 5.53	[5]
	5.0 μm	DWCNTs	3.3	[92]
	15 to 100 μm	SWCNTs	0.15	[16]
	0.2 to 0.6 μm	SWCNTs	0.12	[54]

## Data Availability

Not applicable.
